# The hemostatic effect of hot saline irrigation in endoscopic sinus surgery: a systematic review and meta-analysis

**DOI:** 10.1186/s13643-022-02113-0

**Published:** 2022-11-18

**Authors:** Darshini Nagarajah, Yee Cheng Kueh, Norhafiza Mat Lazim, Baharudin Abdullah

**Affiliations:** 1 Department of Otorhinolaryngology-Head and Neck Surgery, School of Medical Sciences, Universiti Sains Malaysia, 16150 Kubang Kerian, Kelantan Malaysia; 2 Unit Biostatistic and Research Methodology, School of Medical Sciences, Health Campus, Universiti Sains Malaysia, Kubang Kerian, Kelantan Malaysia

**Keywords:** Endoscopic sinus surgery, Hot intranasal saline irrigation, Intraoperative bleeding, Surgical field quality

## Abstract

**Background:**

A good control of intraoperative bleeding is key for adequate anatomical visualization during endoscopic sinus surgery (ESS). The objective of this review was to assess the practice of hot intranasal saline irrigation (HSI) in achieving intraoperative hemostasis and good surgical field quality during ESS.

**Methods:**

An electronic search was performed via PubMed, SCOPUS, Google Scholar, and Cochrane from inception to June 2022. The included trials were evaluated according to the recommendations of the Cochrane Handbook for Systematic Reviews. The primary outcome assessed was the intraoperative bleeding score of the surgical field. The mean arterial pressure, duration of the surgery, amount of blood loss and surgeon’s satisfaction score were assessed as the secondary outcomes. The risk of bias for each study was evaluated using the Cochrane risk of bias tool.

**Results:**

A total of 254 records were identified after removal of duplicates. Based on the title and abstract 246 records were excluded, leaving seven full texts for further consideration. Five records were excluded following full text assessment. Three trials with a total of 212 patients were selected. Hot saline irrigation was superior to control in the intraoperative bleeding score (MD − 0.51, 95% CI − 0.84 to − 0.18; *P* < 0.001; *I*^2^ = 72%; very low quality of evidence) and surgeon’s satisfaction score (RR 0.18, 95% CI 0.09 to 0.33; *P* < 0.001; *I*^2^ = 0%; low quality of evidence). The duration of surgery was lengthier in control when compared to HSI (MD − 9.02, 95% CI − 11.76 to − 6.28; *P* < 0.001; *I*^2^ = 0; very low quality of evidence). The volume of blood loss was greater in control than HSI (MD − 56.4, 95% CI − 57.30 to − 55.51; *P* < 0.001; *I*^2^ = 0%; low quality of evidence). No significant difference between the two groups for the mean arterial pressure was noted (MD − 0.60, 95% CI − 2.17 to 0.97; *P* = 0.45; *I*^2^ = 0%; low quality of evidence).

**Conclusions:**

The practice of intranasal HSI during ESS is favorable in controlling intraoperative bleeding and improving the surgical field quality. It increases the surgeon’s satisfaction, reduces blood loss, shortens operative time and has no effect on intraoperative hemodynamic instability.

**Trial registration:**

PROSPERO registration number: CRD42019117083.

## Background

An ideal surgical setting for endoscopic sinus surgery (ESS) is a bloodless operative field allowing smooth dissection within a relatively reasonable duration of time that helps to avert potential major complications [1]. Excessive bleeding is the main obstacle to endoscopic visualization which may lead to inadvertent complications specifically brain injury, orbital or optic nerve injury, and severe bleeding from major vessels in the sinonasal region [2]. To determine the major postoperative complications associated with ESS such as cerebrospinal fluid (CSF) leak, orbital injury, and hemorrhage requiring blood transfusion, a study conducted a large database review involving a total of 62,823 patients [3]. The review revealed CSF leak was encountered in 0.17% of 40,638 patients, orbital injury in 0.07% of 62,823 patients, and hemorrhage requiring transfusion in 0.76% of 58,752 patients. Though one could argue on the relatively low rate of the major complications reported, their devastating effects on patients are undeniable. The complications may not be solely attributed to poor surgical field but it is not hard to imagine the challenges faced by surgeons to perform a safe dissection with the loss of surgical landmarks arising from the excessive bleeding which could lead to such eventualities. Several techniques have been applied to improve the surgical field during ESS. Some of the most commonly used techniques are intraoperative packing with topical vasoconstrictors, use of bipolar diathermy and induced hypotension. Notwithstanding their effectiveness, there are concerns of safety and potential adverse effects. The use of topical vasoconstrictors such as oxymetazoline, cocaine, and epinephrine have been associated with deleterious effects of hemodynamic instability and must be used cautiously in patients with a history of hypertension or ischemic heart disease and children [4]. Nasal mucosal damage, delayed bleeding, and greater pain were associated with the use of diathermy in patients ensuing nasal surgery [5]. The use of hypotensive technique is dependent on the expertise of the anesthetist, requires the use of anesthetic drugs and higher risk of potential adverse effects [6]. These shortcomings call for an alternative option to be employed for the control of hemostasis.

Intranasal hot saline irrigation is best known for the management of epistaxis [7]. Saline irrigation for epistaxis was found to be easy, less painful and less traumatic to the nose than nasal packing [8]. In addition, hot saline irrigation ranging from 40 to 50 °C has been shown to decrease diffuse oozing from sinonasal mucosa and intracranial bleeding from small vessels [8]. Another benefit of saline irrigation is the cleaning up of the endoscopic lens when blurred by bleeding. The hemostatic mechanism of hot saline irrigation is not evident and may be due to mucosal edema causing pressure being exerted on the injured vessel resulting in blood outflow reduction (tamponade effect) [9]. Hot saline irrigation is also thought to accelerate the clotting cascade mechanism in the human body which aids to reduce bleeding [9].

The practice of intranasal hot saline irrigation during ESS has been introduced to reduce intraoperative blood loss, improve the surgical field quality, and shorten the operative time. The aim of this meta-analysis was to evaluate the hemostatic effect of intranasal hot saline irrigation during ESS.

## Methods

The review protocol was registered in the International Prospective Register of Systematic Reviews (PROSPERO) database under registration number CRD42019117083. The method and reporting were based on Cochrane collaboration [10] and the preferred reporting items for systematic reviews and meta-analysis statement [11]. The evaluation was done according to the Grading of Recommendation Assessment, Development and Evaluation (GRADE) guideline [12]. Randomized controlled trials (RCTs) comparing hot saline intranasal irrigation with control were included.

### Search strategy

An electronic literature search was performed using CENTRAL (Cochrane Central Register of Controlled Trials), Scopus, PubMed, and Google Scholar from inception to June 2022. It was conducted using the search terms “hemostasis”, “nasal saline”, “irrigation”, “endoscopic sinus surgery”, “chronic rhinosinusitis” and “intraoperative blood loss” in combination with Boolean operators “AND” or “OR”. No restrictions were made in terms of period of publication, study duration, and language. Only studies having human subjects for investigation were incorporated in the search. We checked the reference list of identified RCTs and review articles to find unpublished trials or trials not identified by electronic searches. We searched for ongoing trials through the World Health Organization International Clinical Registry Platform; *www.clinicaltrial.gov*. The search strategy is outlined in Table 1.

**Table 1:** Search strategy

Databases	Search strategy
PubMed	((hemostasis) AND (nasal saline irrigation)) AND (endoscopic sinus surgery) AND (chronic rhinosinusitis) AND (intraoperative blood loss))
Cochrane central	Hemostasis in Title Abstract Keyword AND nasal saline irrigation in Title Abstract Keyword AND endoscopic sinus surgery in Title Abstract Keyword AND chronic rhinosinusitis in Title Abstract Keyword AND intraoperative blood loss in Title Abstract Keyword
SCOPUS	Hemostasis in Article title, Abstract, Keywords AND nasal saline irrigation in Article title, Abstract, Keywords AND endoscopic sinus surgery in Article title, Abstract, Keywords AND chronic rhinosinusitis in Article title, Abstract, Keywords AND intraoperative blood loss
Google scholar	Hemostasis in With all of the words, nasal saline irrigation in With the exact phrase, endoscopic sinus surgery/chronic rhinosinusitis/intraoperative blood loss in With at least one of the words and Search where my words occur Anywhere in the article

### Study selection

The primary outcome assessed was the intraoperative bleeding score of the surgical field. The mean arterial pressure, overall duration of the surgery, amount of blood loss, and surgeon’s satisfaction score were assessed as the secondary outcomes. Both primary and secondary outcomes were compared for both comparators intraoperatively. The eligibility criteria were RCTs comparing intranasal hot saline irrigation with control in patients undergoing ESS for chronic rhinosinusitis without the use of additional hemostatic agents including topical or systemic vasoconstrictors. Review authors (DN, KYC) screened the titles and abstracts from the searches and obtained full text articles when they appeared to meet the eligibility criteria or when there was missing or incomplete data to assess the eligibility. Attempts were made to retrieve further details directly from the authors when needed. Studies were excluded from the analysis if the outcome of involvement was not clearly stated with quantifiable data or if it was not possible to extract and calculate the appropriate data from the published results. All the reasons for the exclusion were documented appropriately. Any disagreement between review authors were resolved by another author (BA). Similar strategy was used for the quality assessment.

### Data extraction

Data from eligible studies were extracted using standardized forms and were independently checked by two reviewers (DN, KYC). The reviewers independently extracted the trial characteristics (single or multi-center study type), baseline characteristics of the patients (age, gender, pre-operative American Society of Anaesthesiologist (ASA) status, types of surgery), inclusion and exclusion criteria, the description of the intervention (type of anesthesia, mean arterial pressure, and irrigation administration) and outcomes. The primary outcome assessed was the intraoperative bleeding score of the surgical field. The secondary outcomes assessed were the mean arterial pressure, duration of the surgery, amount of blood loss, and surgeon’s satisfaction score. These outcomes were compared between the intervention (hot saline nasal irrigation) and the control.

### Statistical analysis

The data analysis was performed by using Review Manager 5.3 software based on the random-effects model. Multiple studies were weighted by the amount of information they contribute as recommended by the Cochrane handbook [10] and this was assigned automatically following data entry in Revman. For the continuous outcomes, we reported the mean difference (MD) with standard deviation (SD) or, when necessary, standardized mean difference (SMD). We used the SMD as the summary statistic to standardize the results of studies to a uniform scale. In the case of dichotomous outcomes, we calculated the risk ratio (RR) and odds ratio (OR) with 95% confidence interval (CI). We determined the appropriate units of analysis from the included studies. When analyzing multi-armed trials, we combined all relevant experimental intervention groups in the study into a single group and all relevant control intervention groups into a single control group. If we consider one of the arms to be irrelevant, we excluded it from analysis. The risk of bias for each study was evaluated using the Cochrane risk of bias tool.

## Results

### Study selection

**Fig. 1:**
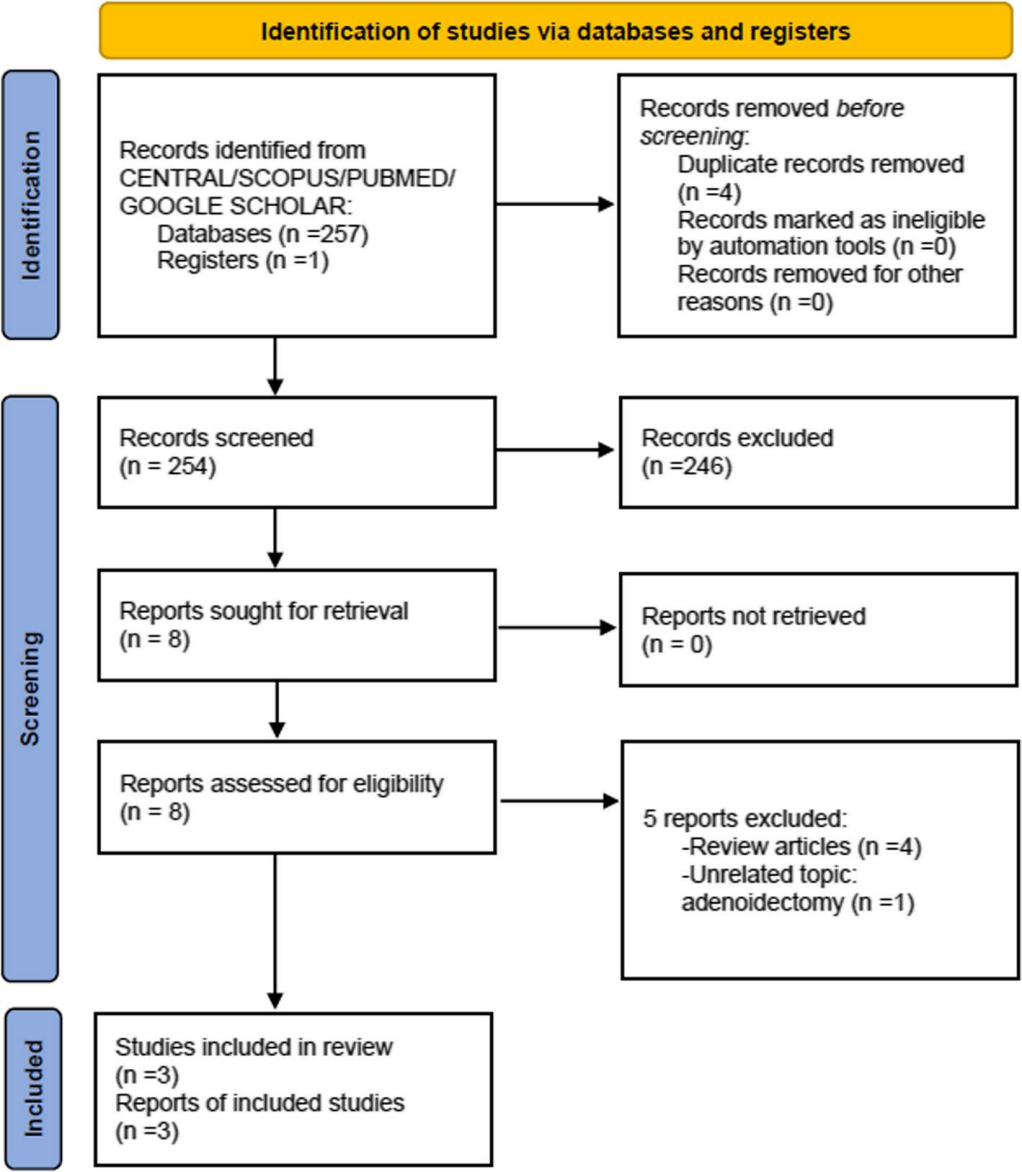
Flow diagram of study selection

A total of 258 records were identified from the searches with 4 duplicate records removed. Two hundred forty-six of the remainder were excluded based on their title and abstract, leaving 8 full texts for further consideration. Eight full-text articles were assessed for eligibility and five records were excluded. A flow chart of study retrieval and selection is shown in Fig. 1. The final analysis included three articles involving 212 patients. The three trials identified were Shehata et al. [13], Al-Ississ et al. [14], and Gan et al [15]. All three trials compared the effects of intranasal saline irrigation with different temperatures in controlling intraoperative bleeding during ESS. All trials recruited adult participants. One of the trials included tranexamic acid as another comparison [13]. Publication bias was not considered because the number of trials included was insufficient to properly assess with a funnel plot.

### Participants

All included trials consist of a total of 212 chronic rhinosinusitis patients who were refractory to medical treatment and underwent surgery. The characteristics of all included trials in the analysis are summarized in Table 2. All trials were single-center trial from different regions namely the cities of Benha (Egypt) [13], Amman (Jordan) [14], and Vancouver (Canada) [15]. Two trials reported the age range for both treatment and control groups from 20 to 58 years [13, 14]. Meanwhile, another trial reported the overall age group was 19 years and above [15]. Both male and female participants were included in the trials [13–15]. All of the patients scheduled for ESS were categorized as ASA class I and II [13–15]. One trial had 3 treatment arms [13] while two trials had 2 treatment arms [14, 15].

**Table 2:** Characteristics of included studies

Author	Study type	Age (years)	Total patients	Intervention	Comparator	Outcomes
Shehata et al. 2014 [13]	RCT	20–50	50	HSI (50 °C)	RTSI (20 °C)	1. Bleeding score is reduced (*p* < 0.001) in HSI (1.96 ± 0.67) compared to RTSI (2.64 ± 0.7). 2. Amount of blood loss is lesser (*p* < 0.001) in HSI (216.25 ± 1.45 ml) than RTSI (272.66 ± 1.78 ml). 3. The duration of surgery is shorter (*p* < 0.001) in HSI (79.22 ± 7.54 min) than RTSI (88.54 ± 8.3 min). 4. Surgeon’s satisfaction score is superior (*p* < 0.001) in HSI (0.88 ± 0.33) compared to RTSI (0.32 ± 0.47). 5. No difference in mean arterial pressure in both groups (*p* > 0.05).
Al-Ississ et al. 2016 [14]	RCT	28–58	100	HSI (48 °C)	RTSI (20 °C)	1. Minimal bleeding score is observed higher (*p* < 0.05) in HSI (80%) than RTSI (48%). 2. Decreased blood loss (*p* < 0.05) occurs in HSI (201.43 ml) compared to RTSI (257.34 ml). 3. HSI has a shorter operative time (*p* < 0.05) of 83.34 min than RTSI of 92.66 min. 4. Surgeon’s satisfaction score is greater (*p* < 0.05) in HSI (88%) than RTSI (32%). 5. No difference in mean arterial pressure of both groups (*p* > 0.05).
Gan et al, 2014 [15]	RCT	≥ 19	62	HSI (49 °C)	RTSI (18 °C)	1. Bleeding score is decreased (*p* = 0.04) in HSI (1.2 ± 0.4) compared to RTSI (1.6 ± 0.6) for long surgical cases (≥ 120 min). 2. Amount of blood loss per minute is reduced (*p* = 0.02) in all cases for HSI (2.3 ± 1.0 ml/min) compared to RTSI (1.7 ± 1.1 ml/min). 3. No difference in mean arterial pressure of both groups (*p* = 0.14). 4. No difference in heart rate of both groups (*p* = 0.32).

*RCT* randomized controlled trial, *HSI* hot saline irrigation, *RTSI* room temperature saline irrigation

### Interventions

Participants in the trials were randomized into either two [14, 15] or three groups [13]. One trial compared hot saline irrigation (50 °C) with room temperature saline irrigation (20 °C) [13], one trial compared hot saline irrigation (48 °C) with room temperature saline irrigation (20 °C) [14] and one trial compared hot saline irrigation (49 °C) with room temperature saline irrigation (18 °C) [15]. Al-Ississ et al. [14] performed the intranasal irrigation using 20 ml hot saline (48 °C) every 10 min, for the entire duration of surgery. Gan et al. [15] administered the nasal irrigation 5 min after the commencement of surgery with 20 ml of hot saline (49 °C) initially and again every 10 min until the end of surgery. Shehata et al. [13] effected 20 ml of intranasal hot saline (50 °C) irrigation, intermittently at regular interval from the start until the completion of surgery. All the trials specified that their patients were induced under general anesthesia with intravenous propofol.

### Risk of bias in included trials

**Fig. 2:**
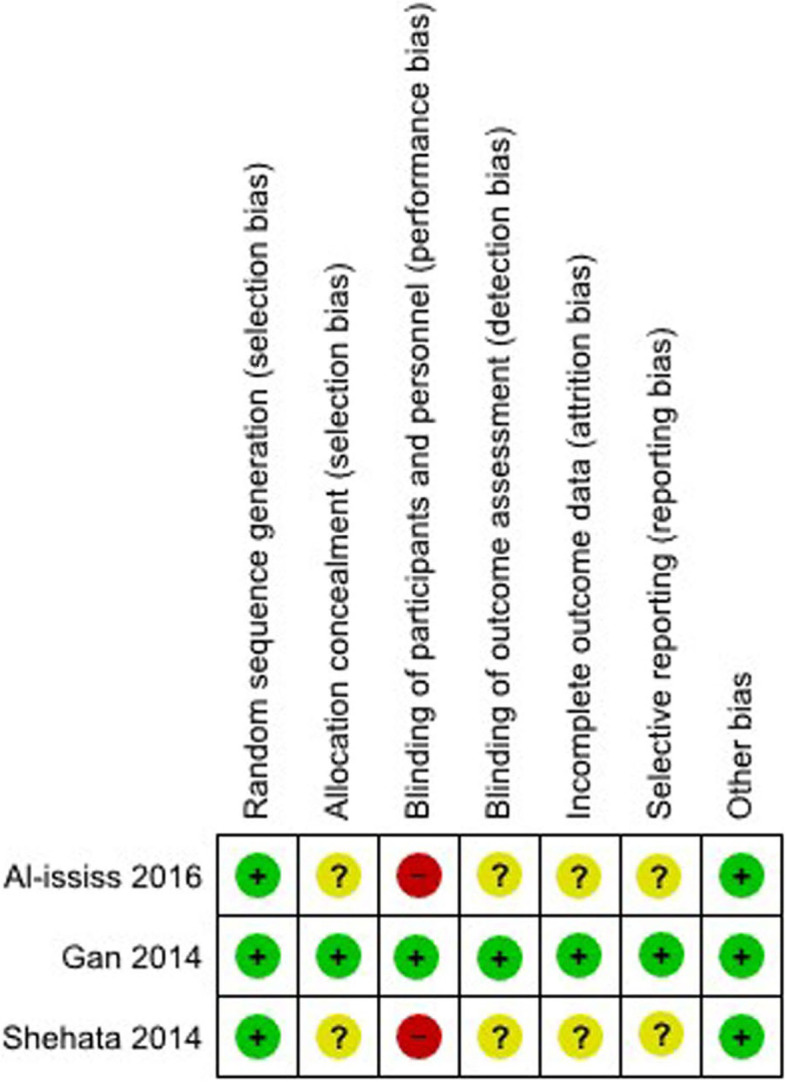
Risk of bias summary of included studies

All three trials had low risk of random sequence generation (Fig. 2). One trial [15] had low risk of allocation concealment as compared to the other two trials [13, 14] which were unclear. Two trials did not report if the participants and the medical personnel were blinded, which constituted a high-risk performance bias [13, 14]. In addition, the detection was unclear in two trials because not all outcomes were blinded [13, 14]. Since it was unclear whether some patients were excluded either pre- or post-intervention, we considered this to be an unclear risk to selection bias. Only one trial [15] registered their protocol while another two trials [13, 14] did not. As we were not able to fully eliminate selective reporting bias in the latter two trials, we appraised them as unclear risk. We did not detect any other possible sources of bias.

### Outcomes

#### Primary outcome

**Fig. 3:**
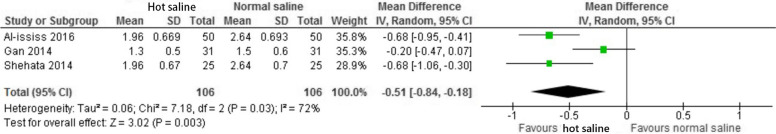
Intraoperative bleeding score

The primary outcome was reported in all three trials [13–15]. All trials [13–15] measured the primary outcome using Boezaart intraoperative bleeding score. Boezaart score is a validated assessment tool for intraoperative bleeding during ESS [16]. It uses a scale from 0 to 5 to evaluate the amount of blood required to be removed intraoperatively to maintain a clear surgical field. Grade 0 is set for a field without bleeding, 1 for slight bleeding without suction, 2 for slight bleeding with suction required, 3 for moderate bleeding which improves for several seconds after suction is removed, 4 for moderate bleeding that occurs immediately when suction is removed, and 5 for severe bleeding that requires constant suction where bleeding occurs more than can be removed. The operating surgeon assessed the intraoperative bleeding score from the start until completion of ESS [13–15]. Hot saline irrigation was found superior to control in the intraoperative bleeding score (MD − 0.51, 95% CI − 0.84 to − 0.18; *P* < 0.001; *I*^2^ = 72%; 3 trials consisting of 212 patients; very low quality of evidence) (Fig. 3, Table 3) [13–15].

**Table 3:** Summary of findings for intranasal hot saline irrigation versus room temperature saline irrigation

Hemostatic effect of intranasal hot saline irrigation during endoscopic surgery					
Patient or population: Chronic rhinosinusitis Setting: Endoscopic sinus surgery Intervention: Hot saline irrigation (48–50 °C) Comparison: Room temperature saline irrigation (18–20 °C)					
Outcomes	Anticipated absolute effects^*^ (95% CI)		Relative effect (95% CI)	№ of participants (studies)	Certainty of the evidence (GRADE)*
	Risk with RTSI	Risk with HSI			
Intraoperative bleeding score	The mean intraoperative bleeding score was **0**	MD **0.51 lower** (0.84 lower to 0.18 lower)	–	212 (3 RCTs)	⨁◯◯◯ Very low^a,b^
Mean arterial pressure	The mean mean arterial pressure was **0**	MD **0.6 lower** (2.17 lower to 0.97 higher)	–	212 (3 RCTs)	⨁⨁◯◯ Low^a^
Surgeon’s satisfaction score	680 per 1000	**122 per 1,000** (61 to 224)	**RR 0.18** (0.09 to 0.33)	150 (2 RCTs)	⨁⨁◯◯ Low^a^
Duration of surgery (minutes)	The mean duration of surgery (minutes) was **0**	MD **9.02 lower** (11.76 lower to 6.28 lower)	–	212 (3 RCTs)	⨁◯◯◯ Very low^a,c^
Amount of blood loss (ml)	The mean amount of blood loss (ml) was **0**	MD **56.4 lower** (57.3 lower to 55.51 lower)	–	212 (3 RCTs)	⨁⨁◯◯ Low^a^

GRADE Working Group grades of evidence

High certainty: we are very confident that the true effect lies close to that of the estimate of the effect.

Moderate certainty: we are moderately confident in the effect estimate: the true effect is likely to be close to the estimate of the effect, but there is a possibility that it is substantially different.

Low certainty: our confidence in the effect estimate is limited: the true effect may be substantially different from the estimate of the effect.

Very low certainty: we have very little confidence in the effect estimate: the true effect is likely to be substantially different from the estimate of effect.

*The risk in the intervention group (and its 95% confidence interval) is based on the assumed risk in the comparison group and the relative effect of the intervention (and its 95% CI).

*CI* confidence interval; *MD* mean difference; *RR* risk ratio; *RCT* randomized controlled trial; *RTSI* room temperature saline irrigation; *HIS* hot saline irrigation

^a^We downgraded the quality of evidence by two due to high risk of performance bias where two trials did not report if the participants and the medical personnel were blinded

^b^We downgraded the quality of evidence by one due to inconsistency in the methodology among the included studies (heterogeneity of 72%)

^c^We downgraded the quality of evidence by one due to imprecision (one study [15], has a small effect and wide CI whereas the other two studies [13, 14] have a very large effect)

#### Secondary outcomes

**Fig. 4:**
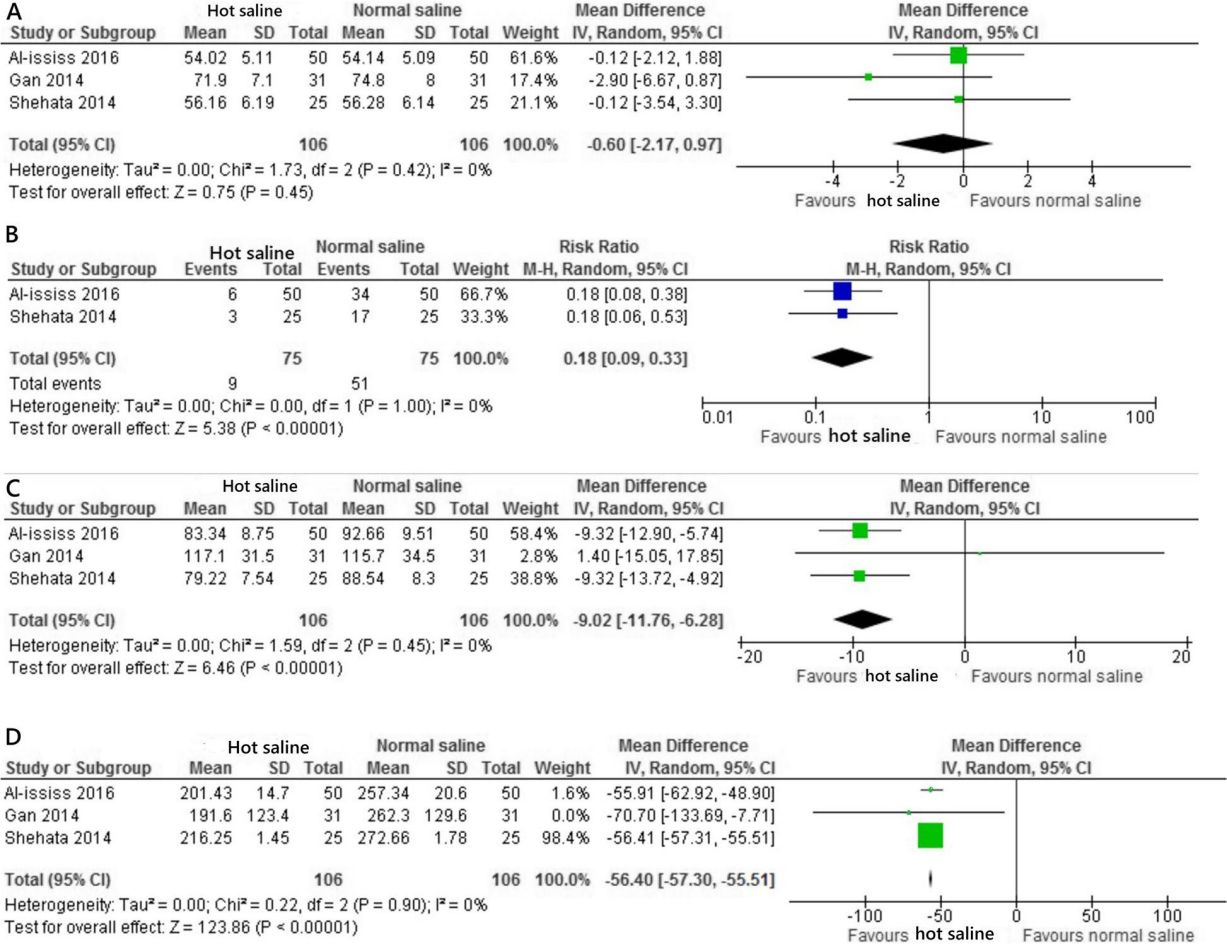
Secondary outcomes. **A** Mean arterial pressure (MAP), **B** Surgeon’s satisfaction score, **C** Duration of surgery (minutes), and **D** Amount of blood loss (ml)

The hemodynamic effect of hot saline irrigation was reflected by the mean arterial pressure. All three trials [13–15] reported the change in mean arterial pressure. The mean arterial pressure was recorded every 10–15 min for the duration of the surgery. There was no significant difference between the two groups for the mean arterial pressure (MD − 0.60, 95% CI − 2.17 to 0.97; *P* = 0.45; *I*^2^ = 0%; 3 trials, 212 patients; low quality of evidence) (Fig. 4, Table 3) [13–15]. Two trials [13, 14] reported the surgeon’s satisfaction score. The score was an assessment to appraise the satisfaction of the surgeon with the surgical field quality which was graded by 5-point Likert scale; 1 = not satisfied at all, 2 = slightly satisfied, 3 = moderately satisfied, 4 = very satisfied and 5 = extremely satisfied [17]. The surgeon’s satisfaction score was significantly improved in hot saline irrigation compared to control (RR 0.18, 95% CI 0.09 to 0.33; *P* < 0.001; *I*^2^ = 0%; 2 trials, 150 patients; low quality of evidence) (Fig. 4, Table 3) [13, 14]. All trials [13–15] reported the duration of surgery. It was measured by the operating time in minutes from the start of surgery until completion. The duration of surgery was significantly lengthier in control when compared to hot saline irrigation (MD − 9.02, 95% CI − 11.76 to − 6.28; *P* < 0.001; *I*^2^ = 0%; 3 trials, 212 patients; very low quality of evidence) (Fig. 4, Table 3) [13–15]. All trials [13–15] reported the amount of blood loss. The estimation of blood-loss volume was measured by subtracting the volume of irrigation fluid from the total volume collected in the suction container in addition to the estimated blood absorbed by the throat pack. The volume of blood loss was significantly greater in control than hot saline irrigation (MD − 56.4, 95% CI − 57.3 to − 55.51; *P* < 0.001; *I*^2^ = 0%; 3 trials, 212 patients; low quality of evidence) (Fig. 4, Table 3) [13–15]. One study reported marked increase in blood loss for the control (257.34 ml) compared to the hot saline irrigation (201.43 ml) [14]. Another study showed blood loss of 191.6 ml in hot saline irrigation compared to 262.3 ml in control [15]. One trial [15] stratified the duration of surgery into short cases (less than 120 min) and long cases (120 min or more) in the assessment of blood loss. In the long cases, greater total blood loss was observed in control (321.1 ml) compared to hot saline irrigation (219.1 ml). One trial reported 4% in the incidence of early postoperative nausea for hot saline irrigation [13]. One study demonstrated no difference in the heart rate (beats/min) in both groups [15].

## Discussion

### Summary of main results

This meta-analysis found the use of intranasal hot saline irrigation provides good hemostatic effect during ESS to control intraoperative bleeding and obtain superior surgical field quality. Hot saline irrigation during ESS prevents excessive bleeding which can compromise the quality of surgical field and avoid complication. There is a close relationship between the intraoperative bleeding and optimal surgical field quality in the outcome of ESS. Good visualizations during ESS are essential and important for complete disease eradication and excessive bleeding during such procedure has been revealed as the one of the reasons for an incomplete surgery [18, 19]. Complications such as damage to orbit or intracranial incursion have been documented when surgical field is compromised by excessive bleeding during surgery [20, 21]. With regard to the temperature of hot saline essential for the hemostatic effect, the present meta-analysis found the range of temperature between 48 and 50 °C was effective. It denotes that this temperature range is possibly ideal to achieve hemostasis. The secondary outcomes of surgeon’ satisfaction score, duration of the surgery, mean arterial pressure, and amount of blood loss were all in favor of the hot saline irrigation. These further reinforces the safety and efficacy of its application in ESS.

In actual practice, multiple preoperative and intraoperative techniques are usually incorporated to improve the surgical field in ESS [22]. Many of them hinge on pharmacological intervention by means of topical vasoconstrictors such as adrenaline or cocaine and systemic vasoconstrictors such as corticosteroids or tranexamic acid. As most of the trials were conducted on healthy individuals without any comorbid conditions, their detrimental effects in high-risk patients are not clear and may be undesirable. In such instances, a non-pharmacological intervention using hot saline irrigation, may perhaps be a good alternative. Moreover, hot saline irrigation in ESS has a distinct hemostatic effect in lengthier surgery which is beneficial for patients with extensive disease such as severe nasal polyposis or complex sinonasal tumor.

### Quality of evidence

All three trials had either low or unclear risk of bias in all six domains except for the performance bias and blinding. Only one trial [15] provided its protocol while two other trials [13, 14] did not, and thus we were not able to fully eliminate selective reporting bias in the latter two trials. However, all trials did report their respective outcomes. Using GRADE, the overall quality of evidence was assessed to be of very low to low quality indicating considerable uncertainty in the estimated effects.

### Agreement and disagreement with other reviews

Up till date, there was one meta-analysis by Ranford et al on hot saline irrigation in ESS [23]. Apart from OVID ® (Wolters Kluwer, N.V), their included databases for literature search was similar to us, with our study having an additional database of SCOPUS (Elsevier, B.V). Notwithstanding the slight discrepancy in the choice of databases, their search found three articles which were the same as ours. In their review, the use of hot saline irrigation during ESS was shown to improve visibility of the surgical field, reduce total blood loss and decrease the operating time. Our review corroborates these findings. In addition, we found the use of hot saline irrigation has no effect on the hemodynamic parameters and beneficial in reducing the intraoperative bleeding. The findings from both studies provide compelling evidence on the benefits of hot saline irrigation and hopefully this would galvanize future studies to be conducted on this subject.

While the advantage of using various topical vasoconstrictors during ESS was demonstrated [4], concerns remain of their harmful effects. It is well acknowledged that the adverse effects of intranasal topical vasoconstrictors are extremely low, thus the minimum sample size used in most trials may not be able to demonstrate such potential detrimental effect.

### Implication for practice and research

Besides being advantageous for protracted surgery such as extensive nasal polyposis and tumor surgery, hot saline irrigation could also be beneficial for endoscopic skull base surgery particularly those involving vascular tumor. Nonetheless, as the effects reflected in primary and secondary outcomes in this meta-analysis are proxies to the clinical outcomes of preventing excessive bleeding and complications such as inadvertent injury to critical structures, the beneficial effects of hot saline irrigation in ESS must be interpreted with caution. The avoidance of complications during ESS with the use of hot saline irrigation could be better understood when more data are available to be scrutinized. Moreover, as different temperatures of hot saline irrigation were applied in the three studies, the ideal temperature to obtain its favorable effects during ESS is unclear. Taken together, these drawbacks indicate further investigations must be done to determine the advantages and disadvantages of hot saline irrigation, before it can be implemented and advocated as a routine practice in ESS.

## Conclusions

The findings of this review suggest that the practice of intranasal hot saline irrigation in ESS is favorable to control intraoperative bleeding and improving the surgical field based on very low to low quality of evidence. Additionally, it shortens the operative time, reduces blood loss, increases the satisfaction of surgeon, and does not cause any intraoperative hemodynamic instability. Owing to the limited trials, further investigations are necessary to confirm these results.

## Data Availability

Not applicable.

## Abbreviations

ESS: Endoscopic sinus surgery
RCTs: Randomized controlled trials
GRADE: Grading of Recommendation Assessment, Development and Evaluation
ASA: American Society of Anaesthesiologist

## References

[1] Alsaleh S, Manji J, Javer A (2019). Optimization of the surgical field in endoscopic sinus surgery: an evidence-based approach. Curr Allergy Asthma Rep..

[2] Al-Mujaini A, Wali U, Alkhabori M (2009). Functional endoscopic sinus surgery: indications and complications in the ophthalmic field. Oman Med J..

[3] Ramakrishnan VR, Kingdom TT, Nayak JV, Hwang PH, Orlandi RR (2012). Nationwide incidence of major complications in endoscopic sinus surgery. Int Forum Allergy Rhinol..

[4] Higgins TS, Hwang PH, Kingdom TT, Orlandi RR, Stammberger H, Han JK (2011). Systematic review of topical vasoconstrictors in endoscopic sinus surgery. Laryngoscope..

[5] Abdullah B, Singh S (2021). Surgical interventions for inferior turbinate hypertrophy: a comprehensive review of current techniques and technologies. Int J Environ Res Public Health..

[6] Barak M, Yoav L, Abu el-Naaj I. (2015). Hypotensive anesthesia versus normotensive anesthesia during major maxillofacial surgery: a review of the literature. ScientificWorldJournal..

[7] Kucik CJ, Clenney T (2005). Management of epistaxis. Am Fam Physician..

[8] Ozmen S, Ozmen OA (2010). Hot saline irrigation for control of intraoperative bleeding in adenoidectomy: a randomized controlled trial. Otolaryngol Head Neck Surg..

[9] Stangerup SE, Dommerby H, Siim C, Kemp L, Stage J (1999). New modification of hot-water irrigation in the treatment of posterior epistaxis. Arch Otolaryngol Head Neck Surg..

[10] Higgins JPT, Thomas J, Chandler J, Cumpston M, Li T, Page MJ (2019). Cochrane Handbook for Systematic Reviews of Interventions.

[11] Moher D, Liberati A, Tetzlaff J, Altman DG; PRISMA Group (2009). Preferred reporting items for systematic reviews and meta-analyses: the PRISMA statement. Ann Intern Med..

[12] Guyatt GH, Oxman AD, Kunz R, Vist GE, Falck-Ytter Y, Schunemann HJ (2008). What is “quality of evidence” and why is it important to clinicians?. BMJ.

[13] Shehata A, Ibrahim MS, Abd-El-Fattah MH (2014). Topical tranexamic acid versus hot saline for field quality during endoscopic sinus surgery. Egypt J Otolaryngol..

[14] Al-Ississ A, Al-Khaldi H, Maayah A, Kilani NK (2016). Effect of warm saline on bleeding during sinus and septum surgery. JRMS..

[15] Gan EC, Alsaleh S, Manji J, Habib AR, Amanian A, Javer AR (2014). Hemostatic effect of hot saline irrigation during functional endoscopic sinus surgery: a randomized controlled trial. Int Forum Allergy Rhinol..

[16] Boezaart AP, van der Merwe J, Coetzee A (1995). Comparison of sodium nitroprusside- and esmolol-induced controlled hypotension for functional endoscopic sinus surgery. Can J Anaesth..

[17] Alimian M, Mohseni M (2011). The effect of intravenous tranexamic acid on blood loss and surgical field quality during endoscopic sinus surgery: a placebo-controlled clinical trial. J Clin Anesth..

[18] Günel C, Sarı S, Eryılmaz A, Başal Y (2016). Hemodynamic Effects of Topical Adrenaline During Septoplasty. Indian J Otolaryngol Head Neck Surg..

[19] Jahanshahi J, Hashemian F, Pazira S, Bakhshaei MH, Farahani F, Abasi R (2014). Effect of topical tranexamic acid on bleeding and quality of surgical field during functional endoscopic sinus surgery in patients with chronic rhinosinusitis: a triple blind randomized clinical trial. PLoS One..

[20] Ceylan SM, Dişikırık İ, Kanmaz MA, Yıldırım A, Sezgin E (2020). Hot nasal packing with hot saline irrigation for hemostasis after adenoidectomy: A prospective randomized controlled study. Int J Pediatr Otorhinolaryngol..

[21] Irita K (2011). Risk and crisis management in intraoperative hemorrhage: Human factors in hemorrhagic critical events. Korean J Anesthesiol..

[22] Acar B, Babademez MA, Karabulut H (2010). Topical hemostatic agents in otolaryngologic surgery. Kulak Burun Bogaz Ihtis Derg..

[23] Ranford D, Fu B, Surda P, Rudd J. Hot saline irrigation for haemostasis in functional endoscopic sinus surgery: a systematic review and meta-analysis. J Laryngol Otol. 2021:1-7. doi: 10.1017/S0022215121003698. 10.1017/S0022215121003698 34819186

